# Supplementary material for: Serotonin modulates insect hemocyte phagocytosis via two different serotonin receptors
Source: eLife. 2016 Mar 14;5:e12241. doi: 10.7554/eLife.12241 (PMC4829436; doi:10.7554/eLife.12241)
Supplement: Supplementary file 1. — DOI: http://dx.doi.org/10.7554/eLife.12241.016 [file elife-12241-supp1.docx]

**Supplementary file 1A Primers used in this research article are presented.**

| **Primer name** | **Primer sequence (5′-3′)** |
| --- | --- |
| **RT-PCR and qRT-PCR for *P. rapae*** | |
| 18s-q-F | CCTGCGGAAGGATCATTAAC |
| 18s-q-R | AACGAAAGAAAGCGTCCAAA |
| TPH-q-F | AAGATTGTACCGGGTTCACG |
| TPH-q- R | AACGATGCCAGTCCAATTTC |
| TRH-q-F | TACCAAAAACACGCCTGTGA |
| TRH-q- R | AGCCAAACCGGAAAGAAAAT |
| SERT-q-F | GCGGCATCACAAATCTTCTT |
| SERT-q-R | TCCGGGTAGACGATGAAAAC |
| 5-HT_1A_-q-F | TTTGCAGCTCAGCATCAATC |
| 5-HT_1A_-q-R | TTGGCTTACGAGGCACTTCT |
| 5-HT_1B_-q-F | AACCTGGTGGGAGACATCTG |
| 5-HT_1B_-q-R | CGGCCAGAGAAAGTATGAGC |
| 5-HT_2A_-q-F | CCAGAGCTATGCGTCATCAA |
| 5-HT_2A_-q-R | TCCACATTGCCTTCTCTGTG |
| 5-HT_2B_-q-F | TCCAGAATGTGAAAAGAGAATACC |
| 5-HT_2B_-q-R | CAATACTGACACAAAAGCACCTT |
| 5-HT_7_-q-F | CTCCGCATCAGAAAAAGCTC |
| 5-HT_7_-q-R | ACGGCGTCAGGAATTGTATC |
| **RACE PCR for *P. rapae*** | |
| TRH-3′outer | ATTGGCAACGCTATATTTCTTCACG |
| TRH-3′inner | CACGGATTCGTTTGAGGAAGCTAAG |
| 5-HT_1A_-3′outer | CCAACGTACAAGGGCCGAAACAATTGC |
| 5-HT_1A_-3′inner | CCACGGTAGACACGTTAGACGAAGAACCA |
| 5-HT_1A_-5′outer | CGCTTCTCATTCCTTATGTGGATGTAGTCG |
| 5-HT_1A_-5′inner | AATCCAACCCTGGCTAACCTCATAAACAGC |
| 5-HT_2B_-3′outer | GGTTGGGTTATGCCTCATCTATGGT |
| 5-HT_2B_-3′inner | TGTAAAAAGAGATAGCCAGCACCCT |
| 5-HT_7_-5′outer | CGCTGTGGAACCTCTTGTAGGGTTC |
| 5-HT_7_-5′inner | GCTGCCCCTCCATTTTTCACATTTA |
| **Complete sequence** | |
| TPH- compF | ACTCTTTCGACCTCTCCTCTC |
| TPH- compR | CACCAATTCACTTTAAACAGCT |
| TRH- compF | CGTCCTTACTCGTCAAACTAACC |
| TRH- compR | CGCCGTTTTGTATGTACTAAAAATT |
| 5-HT_1A_-compF | CGATTTTGAGGAGAGGAGC |
| 5-HT_1A_-compR | AACACATACCGTATCACAGAGC |
| 5-HT_1B_-compF | GATTGTGAGTGAGTGCGATGTGC |
| 5-HT_1B_-compR | GACAGATAACGCCTATTGCCTAC |
| 5-HT_7_-compF | TCAAACGGAGAAACCTGATGTA |
| 5-HT_7_-compR | CTGGGGTATTGACAATCATAGAAAG |
| **Construction of expression vector** | |
| 5-HT_1B_ - KpnⅠ | TT***GGTACCACC***ATGGAGGGCGTGTACC |
| 5-HT_1B_ -EcoRⅠ | TTT***GAATTC***CTAGGGCGGAGCCCGA |
| 5-HT_7_ - KpnⅠ | TT***GGTACCACC***ATGGCGTCTCAAAAT |
| 5-HT_7_ - XholⅠ | CC***CTCGAG***TCATAGAAAGCTCTC |
| **RT-PCR for *D.melanogaster*** | |
| 5-HT_1B_-F | CGTATTCGTAATTGCCGCCA |
| 5-HT_1B_-R | CCGGTCCCAATATCCATCCA |
| 5-HT_2B_-F | ACACCACTGTACAGGAGAGC |
| 5-HT_2B_-R | AGCGGTCTGTCCCCAAAATA |
| TPH-F | TCGAGACCTGGGGCATTATC |
| TPH-R | CCGAGAGCTGAGTAGTCCAG |
| RPL11- F | CGATCCCTCCATCGGTATCT |
| RPL11- R | AACCACTTCATGGCATCCTC |
| **Genomic PCR for *D.melanogaster*** | |
| 5-HT_1B_-genomic-F | CTGCGCTCCTTCTTCAGC |
| 5-HT_1B_-genomic-R | CGTAATTGCCGCCATTATACTC |

**Supplementary file 1B siRNA sequence**

| **Gene name** | **Sense（5'-3'）** | **Antisense（5'-3'）** |
| --- | --- | --- |
| negative control | **UUCUCCGAACGUGUCACGUTT** | **ACGUGACACGUUCGGAGAATT** |
| TPH | **GCUAGCGUUCCGAGUAUUUTT** | **AAAUACUCGGAACGCUAGCTT** |
| Pr5-HT_1B_ | **GCUGACUCCAAGAGAGAAATT** | **UUUCUCUCUUGGAGUCAGCTT** |
| Pr5-HT_2B_ | **CCAGUUCACCAUCAUCUAUTT** | **AUAGAUGAUGGUGAACUGGTT** |
| Pr5-HT_7_ | **GGUUGUGAAGCAUCUCCAUTT** | **AUGGAGAUGCUUCACAACCTT** |
